# Pattern Recognition in Music on the Example of Reconstruction of Chest Organ from Kamień Pomorski

**DOI:** 10.3390/s21124163

**Published:** 2021-06-17

**Authors:** Piotr Wrzeciono

**Affiliations:** Institute of Information Technology, Warsaw University of Life Sciences, 02-776 Warsaw, Poland; piotr_wrzeciono@sggw.edu.pl

**Keywords:** temperament, organ, tonal patterns, climat

## Abstract

The chest organ, which gained popularity at the beginning of the 17th century, is a small pipe organ the size of a large box. Several years ago, while compiling an inventory, a previously unidentified chest organ was discovered at St. John the Baptist’s Co-Cathedral in Kamień Pomorski. Regrettably, the instrument did not possess any of its original pipes. What remained, however, was an image of the front pipes preserved on the chest door. The main issue involved in the reconstruction of a historic instrument is the restoration of its original tuning (temperament). Additionally, it is important to establish the frequency of A4, as this sound serves as a standard pitch reference in instrument tuning. The study presents a new method that aims to address the above-mentioned problems. To this end, techniques to search for the most probable temperament and establish the correct A4 frequency were developed. The solution is based on the modeling of sound generation in flue pipes, as well as statistical analysis to help match a model to the parameters preserved in the chest organ drawing. Additionally, differentalues of the A4 sound values were defined for temperatures ranging from 10 ${}^{\circ }$C to 20 ${}^{\circ }$C. The tuning system proposed in 1523 by Pietro Aaron proved to be the most probable temperament. In the process of testing the developed flue pipe model, the maximum tuning temperature was established as 15.8 ${}^{\circ }$C.

## 1. Introduction

### 1.1. The Reconstructed Instrument

In spring 2014, I was approached by organ builder, Władyslaw Cepka, with a request to help establish the correct temperament for the chest organ from St. John the Baptist’s Co-Cathedral in Kamień Pomorski. The instrument was clearly in a condition that ruled out the possibility of using any standard methods to deal with the problem.

The chest organ is a small pipe organ that is capable of being easily moved. The entire instrument fits into a box (chest), hence its name. Chest organs were popular in the 17th century and were used for basso continuo in chamber music, as well as during religious services.

None of the original pipes from the Kamień Pomorski chest organ have survived. All that remains is an image of the pipes painted on the chest door. On closer inspection, it could be seen that the picture was painted with a fair degree of precision, but as there was no information regarding the pitch of the particular pipes, it was impossible to restore the original temperament with traditional techniques. Another piece of data that was missing was the A4 reference pitch. At present, the pitch standard is 440 Hz. By contrast, the frequencies used back in the 17th century ranged from 392 Hz to approximately 495 Hz (the Arp Schnitger Organ in Hamburg) [1,2]. Another crucial factor to consider was the air temperature at the time the instrument was tuned, as this determines the speed of sound in air as well as the frequency of the acoustic wave generated by a flue pipe.

The 17th century was a time of Little Ice Age [3,4]. The average annual temperatures at the time were approximately 1 ${}^{\circ }$C lower than in 2004. At the same time, the daily temperature fluctuations were considerably greater than today [3]. These factors are reflected in the organ building of the day. Modern pipe organs are tuned at 18 ${}^{\circ }$C or 19 ${}^{\circ }$C [2].

For this reason, when restoring the temperament of a pipe organ, one needs to take into account the following factors: sound generation in a flue pipe, finding the most likely temperament, estimating the frequency of A4 and the most probable air temperature at which the reconstructed instrument was tuned.

### 1.2. Description of the Instrument

**Figure 1:**
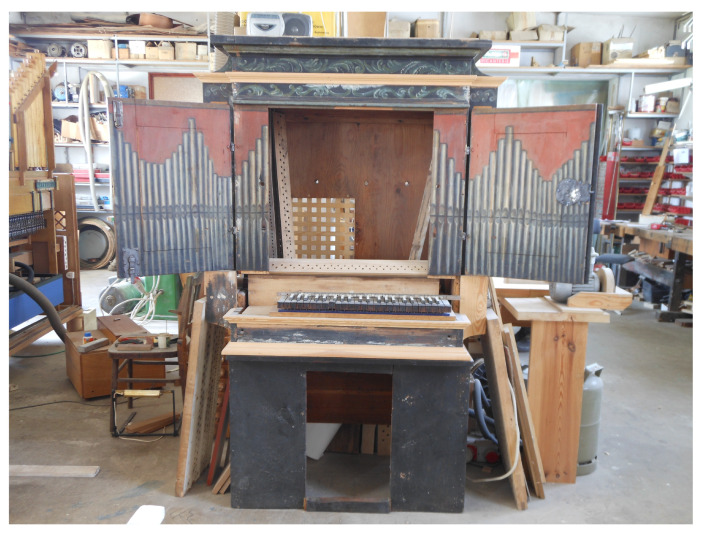
Open chest organ (pipes depicted on the door).

**Figure 2:**
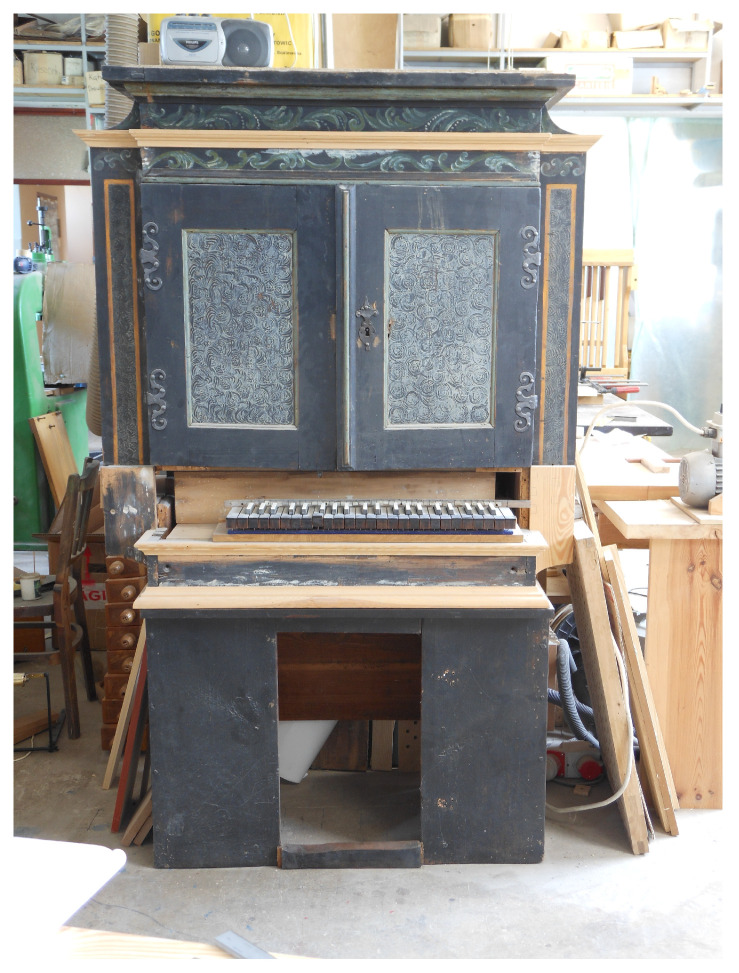
Closed chest organ (with reconstructed keyboard).

**Figure 3:**
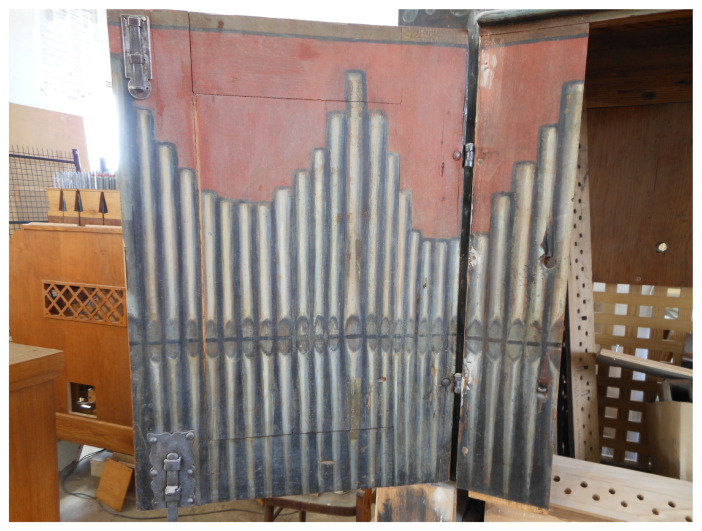
Chest organ left wing.

**Figure 4:**
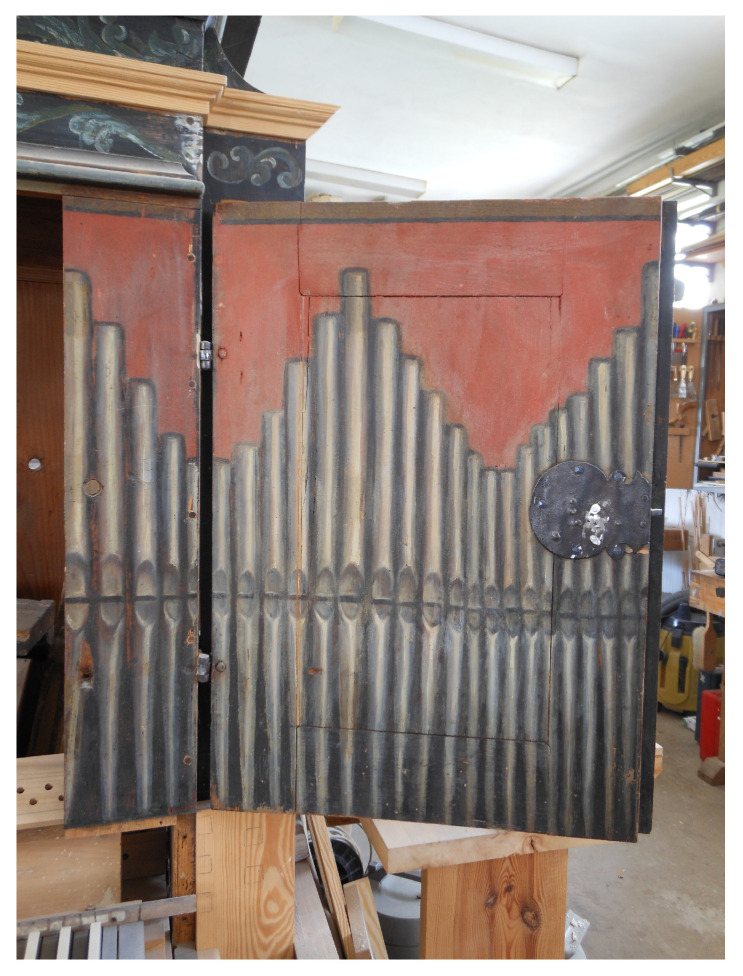
Chest organ right wing.

The chest organ in question dates back to the 17th century. Inscriptions with dates can be found in its windchest (the mechanism that directs compressed air into the pipes). Those include the discernible dates of the instrument’s repairs (1788 and 1829). One can also find an inscription from 1692, which was probably written by the organ builder. What remains of the instrument is its case, featuring an original picture of the pipes, the windchest, stop knobs (controls that turn the required voices on and off), some of the wind ducts carrying air from the bellows compartment to the windchest, and a few keys. Figure 1, Figure 2, Figure 3 and Figure 4 show the instrument as it looked before renovation.

The instrument’s disposition, which was reconstructed based on the inscriptions found on the stops, is as follows: Gedackt 8’, Principal 4’, Octave 2’, Quinte 1 1/3’, Mixture. The disposition of an organ is the structure of its voices (stops). In organ building, a voice is identified by its name as well as its height, expressed in feet. For the 8’ stop, the frequency of the A4 sound is the reference pitch for the whole instrument. Four-foot stops (4’) speak one octave above eight-foot stops (8’). A sixteen-foot stop (16’), in turn, speaks one octave below an eight-foot stop, etc. The pitches of the stops, expressed as a fraction, are used to denote aliquot parts. Aliquots are the harmonics of fundamental frequencies. The most important aliquots include: 1st—the fundamental frequency, 2nd—an octave, 3rd—a fifth, 5th—a third. Quinte 1 1/3’ means the third harmonic of 4’.

The pipes depicted on the chest organ door are open metal labial reeds. The lengths of the “virtual” air columns inside these pipes are shown in Table 1. The measurement of the drawing was performed using a laser caliper. The measurement uncertainty of the device was 0.001 cm. The length of the air column in a pipe is measured from its lower lip to its end [5,6].

## 2. Voicing and Tempering of a Pipe Organ

### 2.1. Sound Generation in Organ Pipes

Sound in an organ pipe is generated by the turbulent air jet at the mouth of the pipe [5,6]. The oscillating air generates an acoustic wave which is reflected from the top end of the pipe. As a result, a standing wave is produced, whose length depends on the length of the pipe. Both the turbulent air jet and the acoustic wave reflection are vital to sound generation in an organ pipe.

Another important parameter is scaling, which is defined as the ratio of an organ pipe’s diameter to its length, measured from the pipe’s lower lip. The larger the scaling, the lower the frequency of a pipe, assuming the air column remains unchanged. This phenomenon has been known for a long time and one can find attempts at its analysis already in the works of Hemholtz [7,8] and Rayleigh [9]. Research into the phenomenon has been largely experimental, as shown in works [10,11], or presented by organ builders, e.g., Astride Cavaillé-Coll “De la Détermination des Dimensions des Tuyaux par rapport à leur Intonation”, Paris, 23 January 1860 [12]. Modern-day research into the phenomena occurring in organ pipes focuses mainly on air flow modeling during sound generation, as well as on the experimental testing of simulation results [13,14,15,16,17,18,19,20,21,22,23,24,25,26,27,28,29,30].

The fundamental frequency of a flue pipe depends, among others, an air temperature. This is because the speed of sound in general depends on temperature (1) [5,6,31].

(1) $$c=\sqrt{\kappa \frac{p}{\rho }}=\sqrt{\frac{C_{p}}{C_{v}}\cdot \frac{RT}{\mu }}$$

In Formula (1), *c* is the speed of sound, κ—the adiabatic exponent, *p*—the static pressure, ρ—the gas density, *R*—the gas constant (R=8.314462175 J/mol · K.), *T*—the temperature [K], μ—the gas molar mass. $C_{p}$ stands for the gas specific heat at a constant pressure, while $C_{v}$ is the gas specific heat at a constant volume. The adiabatic exponent for air at temperature range 0 ${}^{\circ }$C–40 ${}^{\circ }$C is almost constant and equals 1.4 [31]. The value of the molar mass μ of dry air is 0.029 kg/mol.

The relationship shown in Formula (1) is one for an ideal gas [5,6,31]. Air can be treated as an ideal gas in a specific range of pressure and temperature values [6]. In the case of a pipe organ, the pressure is typically expressed as the height of a water column, measured by means of a U–tube manometer. It ranges from 5 to 20 cm of water column [6]. The pressure that is measured with a U-tube manometer is a so-called differential pressure, the values of which are determined relative to the pressure in the wind-chest and the atmospheric pressure. The value of the atmospheric pressure is approximately 1 bar ($10^{5}$ Pa), which is equivalent to 1019.72 cm of water column. The pressure in flue pipes is then only slightly higher than air pressure. For instance, in the case of the differential pressure of 20 cm of water column, the absolute pressure is 1039.72 cm of water column, which is 1.96% higher than normal air pressure. This kind of increase in pressure allows for the application of Formula (1) to the modeling of sound generation in a flue pipe.

Due to the relationships within (1), changes in air temperature result in changes to the fundamental frequency of a pipe, affecting its pitch. These changes are significant from the musical perspective. For example, at 15 ${}^{\circ }$C, an open pipe with an air column length of 0.38646 m generates a sound of 440 Hz fundamental frequency. At 31 ${}^{\circ }$C, the same pipe will have a fundamental frequency of 452.06 Hz.

In order to avoid significant changes in the flue pipe pitch, a few different measures are taken at once. First and foremost, the pipes are supplied with the air from their surroundings, making use of the stable room temperature in relation to the temperature outside. Another solution involves choosing the right season in which to tune the organ. In Europe, this would be the months when temperatures are fairly stable and moderate (April, September). In addition, organ builders use software applications (so-called “organ tuners”) that enable the setting of a required temperature value as an additional parameter. At present, the reference temperature is that of 18 ${}^{\circ }$C [1,2].

Apart from the fundamental frequency, it is also important to arrange the sounds according to a musical scale. This involves the reference frequency A4 and temperament, i.e., the relationships between the particular sounds in a musical scale.

### 2.2. The A4 Frequency in Historic Organs

The current standard pitch of A4 = 440 Hz is classified as ISO 16:1975 [32]. Historically, however, the frequency of a sound was determined arbitrarily, based on the length of a rod or string [33]. Consequently, pitches varied widely across countries and regions. In 1885, A4 = 435 Hz became a standard pitch in Austria [34], already being a standard in France at the time [34]. In the case of historic pipe organs, there is also the question of separate standards for voice and organ, i.e., “choir tuning” (German: Chorton) and “chamber tuning” (German: Kammerton) [33,35], with the chorton pitch being sharp of the kammerton pitch by a semitone. Chorton pitch was primarily used to accompany a choir. Where the organ was also used to play basso continuo, two solutions came into play. One solution involved using kammerton to tune one selected manual, and chorton to tune all the remaining manuals and the pedal [1,36,37]. The other solution involved adding on an extra manual, which would allow the pitch of another manual to be lowered [36,37].

What the ISO 16:1976 [32] standard pitch does not take into account is air temperature. However, this is important in the case of pipe organs, as the standard pitch can only be determined with reference to specific temperature. Instruments that originated in the 17th century were built during the so-called Little Ice Age period [3,4], during which the average annual temperature was 1 ${}^{\circ }$C lower than today [3,4]. In general, temperatures in Europe were considerably lower at the time [3]. For this reason, it would be reasonable to assume that the temperature at which instruments were originally tuned was lower than 18 ${}^{\circ }$C.

Temperature measurement (as we know it today) was first carried out in the 17th century (Giuseppe Biancani, Francesco Sagredo, Christiaan Huygens) [38]. Despite there being no systematic records of temperatures at the time, we can infer the relevant data, mainly on the basis of written accounts, paintings, prints and largely accurate descriptions related to farming (crops and tree growth) [3,39,40]. By analyzing these descriptions, one can reconstruct the temperature patterns during the Little Ice Age period [3].

Based on the study [3], we can accept that, like today, the smallest temperature fluctuations occurred in April and September. At the time that the reconstructed instrument was built (1692), the estimated average temperature was probably 3 ${}^{\circ }$C lower than it is today [3]. The average temperature for September at the time is estimated to be approximately 1${}^{\circ }$ lower than today [3]. It can then be assumed that the temperature at which the reconstructed chest organ was built was in the range of 15–17 ${}^{\circ }$C.

The fact that an organ was originally tuned at a lower temperature may pose considerable problems when performing choral music today. This can be addressed by adjusting the flue pipe length. For example, in the UK, the practice of lengthening all the pipes of a historic organ in order to meet the current standards is not uncommon [41].

### 2.3. Historic Temperaments

Today, the most common tuning system is that of an equal temperament in which the frequency interval between every pair of adjacent notes is always 100 cents. Musical intervals are often expressed in cents. The cent is a logarithmic unit of measure described by Formula (2) [42].

(2) $$I_{M}=1200\log_{2}\left(\frac{f_{1}}{f_{2}}\right)=1200\log_{2}\left(\frac{\lambda_{2}}{\lambda_{1}}\right)$$

In Formula (2), $I_{M}$ stands for the number of cents, while $f_{1}$ and $f_{2}$ are the respective sound frequencies. Instead of sound frequencies, wavelengths for sounds 1 and 2 ($\lambda_{1}$ and $\lambda_{2}$) can also be used.

The principles of equal temperament, which were formulated by Simon Stevin in his manuscript “Vande Spiegheling der singconst”, date back to 1605 [43]. The manuscript was not published until 1884, when fairly similar temperaments were already in common usage. Equal temperament is a system that requires a good, stable frequency pattern because intervals whose frequency ratio is not an integral power of 2 are expressed as an irrational number. The first sufficiently accurate standards were not devised until 1917 [44]. However, it was not until electronic tuners came into use that equal temperament could ultimately be obtained. Such devices guaranteed a sufficiently high degree of frequency stability, without which instruments could never be tuned with a high enough precision [45]. The most significant breakthrough, though, came with the advent of digital technologies in music, especially the appearance of MIDI in 1983 [46]. Regarding this, equal temperament is considered to be a modern invention.

All the tunings that were in use before equal temperament are referred to as historical temperament. There are several dozen temperaments [35,47], all divided into a few groups, depending on the time they were devised or used.

The oldest group comprises temperaments derived from the Pythagorean scale. In this kind of tuning, all the frequency ratios are expressed as rational numbers. However, due to the method employed to calculate the ratio of successive sounds [5,33,48], the octave in this temperament slightly exceeds the value of 2. The difference between the perfect octave (frequency ratio 2) and the Pythagorean octave is defined as the comma. The comma has a value of 23.46 cents (from C to C) [5,33,35,48,49].

Another group, called meantone temperament, results from an attempt to eliminate the above-mentioned difference. The most representative of this group is Pietro Aaron’s temperament, published in Venice in 1523 under the title “Toscanello de la musica” [33,50]. The tuning system described in this work became very popular in Europe, especially during the Renaissance and early Baroque periods [33,35]. The system, however, has a significant drawback: the AS-ES fifth consonance is perceived as a strong dissonance. This interval became known as the “wolf fifth” [5,33,35,48].

The exclusion of the wolf fifth led to the development of “well temperament”. Despite its irregularity, well-temperament does not contain the wolf interval. However, because of its numerous schemes, this tuning system produced a whole family of different temperaments. J.S. Bach’s “Das Wohltemperierte Klavier”, featuring a collection of compositions in all the major and minor keys, is an example of a search for this kind of tuning. Presumably, Bach’s drawing on the collection’s title page illustrates the proportions between intervals [51,52].

Another group of tuning methods comprises near-equal temperaments, including the type of tuning, based on the methods described in the 1840 English publication “Tuner’s Guide #1, #2 and #3” [47]. These were widely used in the second half of the 19th century. As these temperaments were only marginally different from equal temperament, many 19th century pipe organs were adjusted to the latter, usually during restoration works.

The Table 2, based on the work of Jorgensen [47], shows a selection of different temperaments.

The issues discussed here are vital for establishing the correct data regarding the temperament and tuning of the reconstructed chest organ. To find the appropriate temperament, one needs a well-defined set of temperaments. Data concerning temperature are also necessary to estimate the frequency of the sound A4.

## 3. Finding the Pattern of Temperament—Method

### 3.1. Introduction

The original pipes from the chest organ in Kamień Pomorski no longer exist, so there is no possibility of measuring their fundamental frequencies. However, the temperament for the instrument can still be found by means of methods derived from probability theory and statistics. Thus, the first step involved defining the set of possible outcomes and the probability that the temperament computed from the “virtual” pipes (Table 1) is the temperament being sought.

Owen Jorgensen’s publication [47], listing several dozen temperaments from the world of European music, served as a source of possible temperaments. Such a large set of various temperaments provides a good opportunity to test the method employed to find the right temperament for the reconstructed chest organ.

There is a set of temperaments that are very much alike (e.g., those belonging to the set of near-equal temperaments). Additionally, the fundamental frequency of “virtual” pipes can be determined only as a rough approximation, as no information exists about the pressure and velocity of the air flow in the reconstructed instrument.

### 3.2. Set Ω of Possible Temperaments

Every temperament is a set of intervals, expressed in cents. Let us mark this set as $T_{M_{k}}\;$. $T_{M_{k}}$ denotes temperament $T_{M}$ with the index k. Thus, $T_{M_{2}}$ denotes a temperament with the index 2. The set of temperaments also included equal temperament and Lehman’s Bach temperament [51,52]. This gave 82 $T_{M_{k}}$ sets, each of which had intervals defined with reference to sound A. This is made up of 144 intervals and includes all the possible intervals within an octave. The set of possible temperaments consists of $T_{M_{k}}$ subsets and is described by Formula (3).

(3) $$\Omega \;=\;\left\{T_{M_{1}},T_{M_{2}},\ldots ,T_{M_{82}}\right\}$$

### 3.3. Input Set for Temperament Search

The construction of the input set was determined by the $T_{M_{k}}$ and Ω sets. It was decided that all the intervals in such a set must be normalized to an octave. For *N* pipes, the number of intervals would be $N^{2}$, and an interval is calculated with Formula (4).

(4) $$M_{j,k}=\;\left|1200\log_{2}\left(\frac{f_{j}}{f_{k}}\right)\right|\;\mathrm{mod}\;\;1200$$

$M_{j,k}$ denotes an interval between frequencies $f_{j}$ and $f_{k}\;$. The fundamental frequency for open pipes is computed using basic relations (5) [5,6]. As mentioned before, this is an approximate relation, but for pipes that are relatively narrow, the difference between the computed and measured frequencies is insignificant. Due to the small difference, the computed frequency is referred to as the expected frequency. The expected frequency is computed using Formula (5) for an open pipe and Formula (6) for a stopped pipe. The analysis does not include half-stopped pipes (e.g., ones with a soldered smaller pipe), because it would require complex modeling to determine the fundamental frequency [22].

(5) $$f_{\mathrm{open}}=\frac{c}{2L}$$

(6) $$f_{\mathrm{stopped}}=\frac{c}{4L}$$

Formulas (5) and (6), $f_{\mathrm{open}}$ denote the fundamental frequency of an open pipe, $f_{\mathrm{stopped}}$ denotes the fundamental frequency of a stopped pipe, *L* stands for the length of the air column in a pipe, and *c* stands for the speed of sound calculated using Formula (1). In order to construct an input set that will be used in the search for the temperament, it is sufficient to take the length of the air column in a pipe and its type (open or stopped) into account.

Thus, the input set is a set of intervals $\Xi =\{M_{1,1},M_{1,2},...,M_{N,N}\}$ computed solely on the basis of the length of the air column for *N* pipes, using Formulas (4)–(6).

### 3.4. Probability of Temperament

The probability of a temperament is a measure of similarity between the Ξ temperament input set and a temperament in the Ω set. The number of elements in the Ξ set may differ from 144 (the number of intervals in $T_{M_{k}}$ sets). In addition, intervals in the Ξ set are subject to measurement uncertainty, whereas those in $T_{M_{k}}$ are assumed to be perfect, since they were devised as a kind of proportion [5,42,47]. Consequently, measurement uncertainty (expressed as $\sigma_{\mathrm{cent}}$—uncertainty of musical interval) needs to be taken into account when searching for the desired temperament. The value of the error needs to be predetermined, or determined heuristically.

The probability that set Ξ is identical to a given $T_{M_{k}}$ set is defined as follows:

*W* is the number of distinguishable intervals in set Ξ that match the intervals in set $T_{M_{k}}$ with a $\sigma_{\mathrm{cent}}$ accuracy. Distinguishable intervals are those that differ from one another by more than $\sigma_{\mathrm{cent}}\;$. Unisons are not taken into account. Consequently, the number of distinguishable intervals in set Ξ that match intervals in set $T_{M_{k}}$ equals 132. Accordingly, $p_{k}\;$, i.e., the probability that set Ξ matches the temperament of $T_{M_{k}}$ is as follows (7):

(7) $$p_{k}=\frac{W}{132}$$

In Equation (7), $p_{k}$ is the probability calculated for the *k*th temperament (in $T_{M_{k}}$).

With the assumed measurement uncertainty $\sigma_{\mathrm{cent}}\;$, we obtain a set of probabilities $\left\{p_{1},p_{2},...,p_{82}\right\}$ which are then arranged in descending order. The temperament with the highest probability can be regarded as the correct temperament. However, there is a risk that when the value of $\sigma_{\mathrm{cent}}$ is too high, we will get several temperaments with a probability of 1. There is also another possible scenario where the highest probability is 0.5 or less, which makes it impossible to decide which temperament is correct. A situation like this is possible when the number of remaining pipes is too small.

When several probabilities $p_{k}$ have the same value = 1, it is possibly because the assumed uncertainty of the temperament was too high. This means that there is a considerable number of temperaments that are very similar [47]. The problem was solved by introducing another measurement of similarity between the set of intervals obtained from the pipes and the intervals in $T_{M_{k}}$. This is the number of intervals obtained from the pipes that match the intervals in $T_{M_{k}}$. The number was marked as $P_{M_{k}}\;$, and is computed for every temperament from the set Ω.

### 3.5. Algorithm of Searching for the Most Probable Temperament

The procedure is as follows:

1. Create set Ξ;
2. Create set Ω;
3. Specify the initial value of $\sigma_{\mathrm{cent}}\;$;
4. Specify the step of interval uncertainty reduction $\Delta \sigma_{\mathrm{cent}}\;$;
5. Compute set $\{p_{1},p_{2},...,p_{82}\}\;$;
6. Does $\left\{p_{1},p_{2},...,p_{82}\right\}$ contain at least two values of 1?;
7. If so, $\sigma_{\mathrm{cent}}\leftarrow \sigma_{\mathrm{cent}}-\Delta \sigma_{\mathrm{cent}}\;$, then go back to 5;
8. For each temperament in Ω, calculate the value of $P_{M_{k}}\;$;
9. Sort $\left\{p_{1},p_{2},...,p_{82}\right\}$ in descending order (in relation to $P_{M_{k}}$ values);
10. Return a well-ordered set from 9, and the ultimate value of $\sigma_{\mathrm{cent}}\;$.

### 3.6. Graphical Presentation of the Search Result

For the purpose of presenting the overall outcome of the temperament search, the results are shown in the form of a compliance matrix.

The compliance matrix is presented as a table consisting of 12 rows and 12 columns. The columns and rows represent sounds in the twelve-tone scale, whereas the cells represent intervals between the sounds. The figure in each cell denotes the number of consonant intervals between pipes (with the exception of the diagonal, which is not taken into account during calculations). Intervals present both in Ξ and $T_{M_{k}}$ (for a selected *k*) are marked in green. If a given interval is present in $T_{M_{k}}$ but not in Ξ, the cell is marked in red. This kind of graphical presentation allows one to see which temperaments are more compliant and which are less compliant with set Ξ.

The Table 3 shows the best compliance matrix for the reconstructed chest organ from Kamień Pomorski. For example, the cell marked as C#–A contains the number 3, which means that three intervals from Ξ match an interval in $T_{M_{k}}$ (Pietro Aaron’s temperament).

The compliance matrix was created for each of the 82 temperaments. By doing this, it was possible to check which of the intervals from set Ξ are present in each temperament.

Finding the most probable temperament is the first step in the reconstruction of data relating to the tuning of the instrument. Following that, it is necessary to establish the correct frequency of the sound A4, and the air temperature in which the organ was tuned.

## 4. Method of Searching for A4 Reference Pitch—The Second Pattern

### 4.1. Defining the Frequency Boundaries of the Sound A4—Discussion

Scales in music are primarily based on the scale of the human voice [49]. This fact is of great importance when we consider the organ—an instrument that is mainly used to accompany singers. For this reason, there are two types of tuning that have been in common use, i.e., the choir tuning (chorton) and the chamber tuning (kammerton), as mentioned before. Thus, for an organ to be used as an accompanying instrument, its scale has to match the capacity of the human voice.

One important feature connected with the human voice is the stable temperature of the exhaled air [53], which allows for the production of a pitch independent of the air temperature. Obviously, this does not apply to extremely high or low temperatures.

Unlike with singers, the frequency of the sound generated by organ pipes varies according to the changing temperature. This is because the speed of sound depends on temperature (1). Therefore, a search for the frequency of A4 needs to be carried out with reference to the optimal temperature.

Considering the modern values of reference pitch A4—440 Hz for kammerton and 466 Hz for chorton—one has to search through similar frequency bands. It is also necessary to take into account any preserved or reconstructed historical models, where the lowest frequency value of A4 is 392 Hz [1]. This is approximately one whole tone below 440 Hz. The Victorian concert pitch [41] also needs to be considered, where A4 had the value of 453 Hz.

By juxtaposing the historical reference pitches [1] with the reference pitch, A4, used today, we arrive at the data shown in Table 4.

Some extreme values, such as A4 = 495 Hz, are not unknown in organ building, but these are characteristic of instruments constructed during the Little Ice Age period, for instance, the Arp Schnitger organ in Hamburg (1693). The frequency of 495.5 Hz was measured at 18 ${}^{\circ }$C during the instrument’s reconstruction (1990–1993) [2]. It is then possible that the original tuning was carried out at a lower temperature.

Taking the historical frequencies of A4 into account, as well as the influence of air temperature on the speed of sound, it was assumed that that the reference pitch A4 of the reconstructed chest organ should be in the range of 392–495 Hz.

### 4.2. The Method of Searching for A4

The values of measurement of the actual pipe or one depicted on the chest organ door are both subject to measurement uncertainty. Additionally, one has to bear in mind that the fundamental frequency of a pipe depends on air pressure and air velocity. Therefore, it is to be expected that, despite the stablishment of a fairly accurate temperament, the measurement uncertainty of the A4 sound frequency will be higher. For this reason, the parameter $\sigma_{\mathrm{freq}}\;$, expressed in cents, was introduced. This kind of error needs to be assumed at the start, and possibly reduced later on. $\sigma_{\mathrm{freq}}$ can be interpreted as follows: it is a space (interval) around a frequency that is regarded as equivalent to the frequency of a given sound.

Another important notion is the set of chromatic scales. A pipe organ comprises stops from 32’ to 1’, i.e., from C0 to C10 (10 octaves). 64’ stops, e.g., the Henry Willis organ of Liverpool Cathedral (1923–1926), as well as several stops exceeding 64’ have been left out because these sounds are perceived as vibrations. Consequently, the full musical scale of a pipe organ includes 10 octaves.

The lowest possible frequency $f_{A_{\min}}$ of A4, the maximum frequency $f_{A_{\max}}\;$, and the step of frequency change in the search for $\Delta f_{A}$ are some other concepts that will also be needed in our search for the desired frequency.

For a specified temperament and A4 frequency, a set containing all the frequencies within the scale (from C0 to C10) is created. The set is denoted as $S_{f}\;$, where f is given the value of A4 reference pitch, e.g., $S_{435}$ would be a set of frequencies of a full scale (10 octaves) calculated for A4 = 435 Hz.

In order to find the correct temperament, we need an additional set, that will contain different values of $S_{f}\;$. The set is defined by Formula (8):

(8) $$\Gamma =\;\left\{S_{f_{1}},S_{f_{2}},S_{f_{3}},\ldots ,S_{f_{z}}\right\}$$

Γ denotes a set of musical scales. The particular scales are denoted as $S_{f_{1}},S_{f_{2}},S_{f_{3}},\ldots ,S_{f_{z}}$, where $f_{1},f_{2},f_{3}$ and $f_{z}$ are the successive reference frequencies of A4.

Additionally, a set $P_{S}$ was introduced, containing the frequencies of organ pipes calculated for the specified temperature T.

Essentially, the search for the fundamental frequency of A4 of the reconstructed instrument means finding a subset $S_{f}$ within set Γ, which would contain the largest number of frequencies from set $P_{S}\;$, with the specified tuning uncertainty $\sigma_{\mathrm{freq}}\;$. The algorithm for searching for the fundamental frequency of A4 will then be as follows:

1. Specify $T,f_{A_{\min}},f_{A_{\max}},\Delta f_{A},\sigma_{\mathrm{freq}}\;$, the set of pipe lengths, and the temperament;
2. Create set $P_{S}$ for air temperature T;
3. Generate set Γ for the specified temperament and frequencies from $f_{A_{\min}}$ to $f_{A_{\max}}\;$, every $\Delta f_{A}\;$;
4. For each set $S_{f}$ within Γ, calculate how many frequencies from each particular set are present in $P_{S}\;$. Remember this value as $H_{f}\;$, where *f* denotes the same frequency as in $S_{f}\;$. The accuracy of the comparison is specified in advance by the interval determined by $\sigma_{\mathrm{freq}}\;$;
5. Specify *f* for the highest $H_{f}$ as the frequency of the reference pitch A4.

### 4.3. Finding the Correct Temperature—The Third Pattern

The reconstructed instrument dates back to the so-called Little Ice Age. Finding the correct tuning temperature is, therefore, of vital importance. The search for the correct temperature was carried out by broadening the range of frequencies, and by making calculations for various temperatures using the method described in Section 4.2 above.

**Figure 5:**
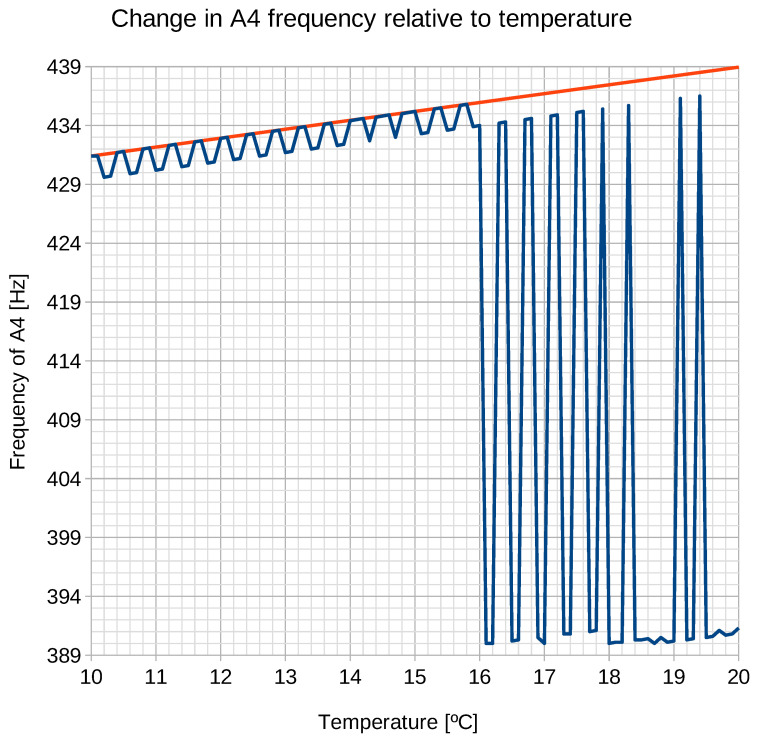
The relationship between the sound A4 frequency (Pietro Aaron’s temperament) and temperature. The change in frequency of pipe A4 of λ = 0.39073 m (435.8 Hz for T = 15.8 ${}^{\circ }$C) is marked in red.

The calculations were made for a frequency range of 380 Hz–500 Hz, for temperatures from 10 ${}^{\circ }$C to 20 ${}^{\circ }$C, with 0,1 ${}^{\circ }$C intervals. The results are shown in Figure 5.

The graph (5) shows a visible change in the upward trend in the frequency of A4 as temperature increases. The color red is used here to mark the frequency of the sound A4 for a pipe of λ = 0.39073 m, taking the relationship between temperature and the speed of sound (1) into account. Up to the value of 15.8 ${}^{\circ }$C, this dependence is consistent with the dependence expressed by Formula (1). The small discrepancies result from the value of $\sigma_{\mathrm{freq}}$ set at 10 cents.

As long as the tendency is consistent with the dependence resulting from thermodynamics (1), the frequency of A4 is correct. When this dependence breaks, there is no basis for a given frequency of A4 to be regarded as correct. Therefore, the chest organ from Kamień Pomorski was tuned at the maximum temperature of 15.8 ${}^{\circ }$C, with a frequency of A4 = 435.8 Hz.

For this frequency of A4, 30% of the depicted pipes fall into a set of frequencies 435.8 Hz with $\sigma_{\mathrm{freq}}$, which does not exceed 10 cents.

## 5. Discussion

### 5.1. Introduction

The methods discussed in Section 3 and Section 4 required further testing on other sets of data in order to verify the returned results. To this end, files with air column lengths calculated for several temperaments, such as equal, Pietro Aaron’s, Valotti’s and Tuners guide well #3 [47], were used. The tests made it possible to specify the minimum amount of data necessary to find the required temperament, as well as to define the relationships needed for the correct establishment of the sound A4 frequency for different temperaments.

### 5.2. Establishing the Minimum Number of Pipes

The number of intervals was determined on the basis of pipes is $N^{2}-N\;$, where *N* denotes the number of pipes of an identifiable size (the height of air column and the wave length). For each of the temperaments used in the search, the number of possible intervals was 132. Consequently, the minimum number of pipes of different length is 12. However, this assumption only holds true if we can be certain that the pipes in question are capable of forming all the intervals in the temperament.

**Figure 6:**
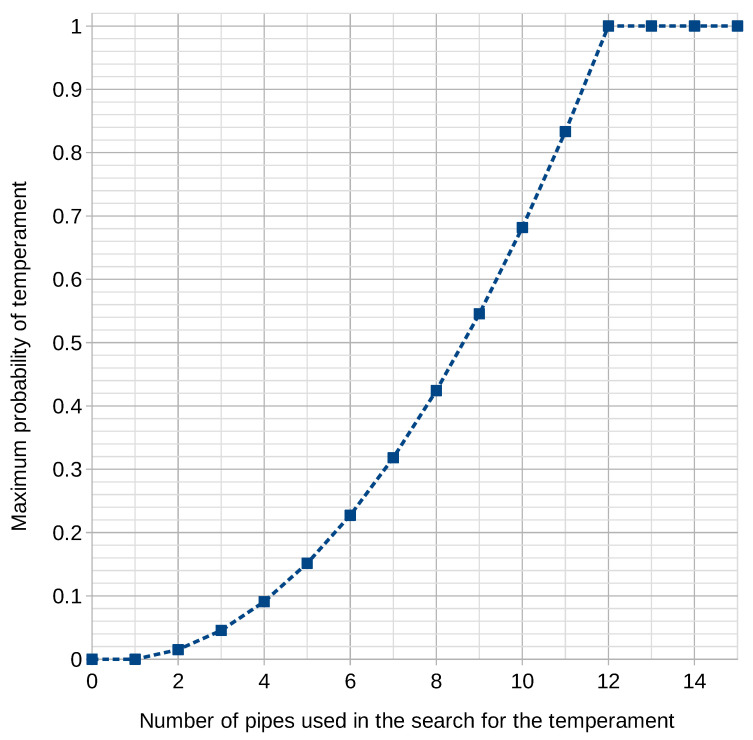
The maximum probability of finding the temperament relative to the number of pipes used in the search for the required temperament.

The graph (Figure 6) shows the relationship between the highest possible probability and the number of pipes used to establish the temperament.

What constitutes an additional restraint is the fact that the pipes being analyzed are usually representative of the whole instrument. Therefore, the representativeness of the sample is also a restraint. As an organ is made up of a number of pipes, one has to ensure that the representative sample is a large random sample. In statistical analysis, N≥30 can be used as a large sample size [54].

In the case of the reconstructed instrument, 50 pipes were painted on the chest door. This is considerably more than 12, and also more than 30, which fulfills the requirements of statistical analysis [54], and meets the required condition for the correct functioning of the algorithm used in the search for the temperament.

In conclusion, the methodology used in mathematical statistics suggests that the minimum number of pipes with varying air column lengths has to be equal to or larger than 30.

### 5.3. Correct Method of Establishing the Frequency of A4 Sound

The correlation was tested using the following temperaments: equal, Pietro Aaron’s, Valloti’s and Tuners guide well #3. Each of these is very important in music. Equal temperament is commonly used today. The temperament developed by Pietro Aaron remained in use for at least 200 years. Other unequal temperaments are mere modifications of Aaron’s temperament. Valotti’s temperament is widely used by chamber ensembles specializing in so-called historical music performance. Tuners guide temperaments had an enormous influence on piano tuning. The #3 temperament can be found across Europe, because it is not too different from teh equal temperament.

The testing was carried out on computed sets (four octaves, from C1 to C5), which were both accurate and randomly modified (up to 10 cents). Pietro Aaron’s temperament proved insensitive to such measurement uncertainty, whereas, in the case of Valotti’s temperament, the obtained frequency of A4 was sometimes different (by up to 2 Hz) from the original. With equal temperament and Tuners guide well #3, the maximum difference between the preset testing frequency and that obtained was 0.3 Hz.

In the case of equal temperament and Tuners guide well #3, there was also another problem that occurred in connection with the range of frequencies being searched. When the frequency spectrum was wider by more than one semitone (e.g., A4 and G#4), the returned fundamental frequency was lower. This is mainly due to the fact that, when intervals are an integer multiple of 100 cents, the reference frequency can have any value as long as it matches the scale.

Therefore, when it becomes apparent that the temperaments being dealt with are nearly equal, the search range should be narrowed down to 433 Hz–456 Hz. This particular range comes from the A4 frequencies that were used at the time when near-equal temperaments were already in use [33,47].

### 5.4. Establishing the Tuning Temperature

Establishing the correct tuning temperature is particularly important when dealing with a period in history for which there are no reliable meteorological data. This is always necessary for a baroque pipe organ.

The temperature obtained for the Kamień Pomorski chest organ (15.8 ${}^{\circ }$C) coincides with the data that can be found in publications related to that particular period (April 1692) [3]. It seems reasonable to assume that historic pipe organs can also be used as a further source of data concerning temperature. We need to remember, however, that those instruments were used indoors, where the temperature changes less quickly than outside.

## 6. Final Results

Thanks to the developed method, the following data were obtained regarding the temperament and tuning of the chest organ from Kamień Pomorski (Table 5 and Table 6):

For comparative purposes, the ten best temperament search results and A4 frequencies are shown in Table 7.

Following the reconstruction of the temperament, the sound itself was reconstructed by means of Grand Orgue. Using samples, a virtual instrument was created whose disposition was identical to that of the reconstructed chest organ. Afterward, the temperament of the reconstructed organ was adjusted to the value established in the study (Table 5), and the A4 pitch was lowered from 440 Hz to 435 Hz (by 19 cents). This allowed for a musical test of the instrument before it was physically reconstructed. It emerged that, despite its modest disposition, the instrument has an interesting tone color, which is very appropriate for the performance of early baroque music.

## 7. Conclusions

The presented method of searching for the correct temperament and reference pitch of the A4 sound can be used more widely for the renovation or reconstruction of a historic pipe organ. This, however, is conditioned on the minimum number of pipes used in the search, i.e., a minimum of 30 pipes. One important feature of the developed method is that it offers the possibility of looking at different solutions that are feasible from the historical perspective.

It is also important that the method is effective, regardless of whether the pipes carry a mark that assigns them to a particular sound of a given voice.

Establishing the correct air temperature at which an instrument was originally tuned is critical to the renovation of the instrument. Quite possibly, due to a lack of similar methods, a large number of instruments have been unwittingly tuned too high. By applying the presented methods, one can avoid a mistake which could irreversibly damage a historic instrument.

The possibility of establishing the original tuning temperature of a historic organ could also have a practical application in studies concerning climate change, by aiding the existing methods of historical data analysis of a period when temperature could not be recorded.

However, the application of the discussed methods has its constraints. Firstly, the number of pipes that make up a representative sample to form an input set for the search should be no less than 30. In addition, one has to ensure that the sample includes pipes of varying length.

Besides, different temperaments display different sensitivities to the measurement uncertainty of the input set. Therefore, when the temperaments are similar, one has to decide which temperament is more likely from the historical perspective. This seems to mainly be important for instruments that might have already been tuned with a near-equal temperament.

## Figures and Tables

**Table 1:** Measurements of the “virtual” air column length in the pipes of the chest organ door.

Left Wing		Right Wing (Numbering Continued)	
**Pipe Number, from Left to Right (Figure 1 and Figure 3)**	**Air Column Length [cm]**	**Pipe Number, from Left to Right (Figure 1 and Figure 4)**	**Air Column Length [cm]**
1	34.850	26	32.250
2	28.615	27	27.225
3	24.515	28	21.830
4	21.620	29	16.615
5	18.975	30	13.990
6	18.040	31	14.240
7	17.900	32	15.995
8	17.730	33	19.055
9	19.445	34	24.240
10	21.850	35	28.680
11	24.520	36	33.016
12	29.200	37	28.030
13	34.190	38	24.640
14	29.400	39	21.240
15	24.510	40	17.900
16	19.750	41	15.580
17	14.500	42	14.155
18	13.350	43	14.070
19	13.145	44	13.905
20	13.645	45	18.425
21	14.645	46	20.400
22	19.715	47	21.630
23	23.510	48	24.930
24	27.905	49	27.705
25	32.410	50	33.725

**Table 2:** Examples of temperaments.

Type of	Intervals [Cents]											
Temperament	A–A	A–A#	A–B	A–C	A–C#	A–D	A–D#	A–E	A–F	A–F#	A–G	A–G#
Equal	0.00	100.00	200.00	300.00	400.00	500.00	600.00	700.00	800.00	900.00	1000.00	1100.00
Pythagorean	0.00	90.23	189.18	294.14	393.09	498.05	597.00	701.95	792.18	891.14	996.09	1095.05
Pietro Aaron	0.00	117.00	193.00	310.00	386.00	503.00	620.00	696.00	813.00	889.00	1007.00	1083.00
Tuner’s guide well #3	0.00	100.99	199.09	300.35	398.71	500.21	601.07	699.07	800.21	898.47	999.88	1100.65

**Table 3:** Chest organ compliance matrix for Pietro Aaron’s temperament ($p_{k}=0.985$; $\sigma_{\mathrm{cent}}=0.53$ Cent).

	A	A#	B	C	C#	D	D#	E	F	F#	G	G#
A	0	5	3	2	3	3	2	3	3	2	3	5
A#	5	0	3	3	1	3	3	2	3	2	2	2
B	3	3	0	5	3	2	2	3	2	3	3	2
C	2	3	5	0	3	3	2	3	3	2	3	2
C#	3	1	3	3	0	5	2	2	2	3	2	3
D	3	3	2	3	5	0	5	3	2	3	3	2
D#	2	3	2	2	2	5	0	3	3	1	3	0
E	3	2	3	3	2	3	3	0	5	3	2	3
F	3	3	2	3	2	2	3	5	0	3	3	1
F#	2	2	3	2	3	3	1	3	3	0	5	3
G	3	2	3	3	2	3	3	2	3	5	0	3
G#	5	2	2	2	3	2	0	3	1	3	3	0

**Table 4:** Musical sounds used today juxtaposed with historical frequencies of A4.

Frequency	392 Hz	415 Hz	435 Hz	440 Hz	466 Hz
Musical sound	G4	G#4	A4	A4	A#4

**Table 5:** Reconstructed temperament of the Kamień Pomorski chest organ.

Temperament	Meantone, 1/4–Comma, Published by Pietro Aaron	
Temperament probability:	0.985	
Frequency of A4:	435.2 Hz	435.8 Hz
Tuning temperature of the instrument:	15.0 ${}^{\circ }$C	15.8 ${}^{\circ }$C

**Table 6:** Frequencies of notes in the middle octave at 15.0 ${}^{\circ }$C.

Note	C4	C#4	D4	D#4	E4	F4
Hz	260.31	272.00	291.04	311.40	325.39	348.16
**Note**	**F#4**	**G4**	**G#4**	**A4**	**A#4**	**B4**
Hz	363.80	389.25	406.74	435.20	465.66	486.57

**Table 7:** Ten best temperament search results.

Order	Temperament	Probability $p_{k}$	Value of $P_{M_{k}}$	A4 frequency [Hz] for 15 ${}^{\circ }$C
1	Pietro Aaron quarter syntonic (1523)	0.985	364	435.2
2	Charles Earl Stanhope (1806)	0.864	356	433.3
3	John Holden 1/5 syntonic (1770)	1.000	354	434.0
4	Marin Mersenne & Lambert Chaumont (1636)	0.955	354	433.2
5	Improved William Hawkes meantone (1798)	0.970	348	434.0
6	1797 meantone	0.970	344	434.0
7	1799 meantone	0.970	344	434.0
8	William Hawkes meantone (1798)	0.985	342	434.0
9	Johann Philipp Kirnberger “well” (1801)	0.970	340	435.3
10	A. Merrick quite equal (1811)	0.955	334	431.8

## Data Availability

All necessary and relevant data are included in this paper.
